# Geospatial quality assessment of locally available ice for heavy metals and metalloids and their potential risks for human health in Karachi, Pakistan

**DOI:** 10.1016/j.heliyon.2024.e28252

**Published:** 2024-03-22

**Authors:** Aamir Alamgir, Qamar Ali, Noor Fatima, Moazzam Ali Khan, Muhammad Farrakh Nawaz, Somia Tariq, Muhammad Rizwan, Jean Wan Hong Yong

**Affiliations:** a Institute of Environmental Studies, University of Karachi, Karachi, Pakistan; b Department of Environmental Sciences, Government College University Faisalabad, Faisalabad, 38000, Pakistan; c Department of Biosystems and Technology, Swedish University of Agricultural Sciences, 23456 Alnarp, Sweden

**Keywords:** Ice quality, Health, Contamination, Risk, Metals, Metalloids

## Abstract

Extreme hot conditions during summers, high poverty rate and continuous electricity load shedding affect commercial manufacturing and sale of ice in many countries. The vendors prepared ice using untreated piped water, tanker water and ground water. These waters may contain hazardous pollutants and ice made from them will pose a potential human health risk. Thus, it is important to regularly monitor the chemical composition of water sources and the quality of the manufactured ice. A contemporary examination was carried out to evaluate the physico-chemical properties and heavy metals and metalloids in the ice sold in all the districts of Karachi, Pakistan. This pioneering study was an innovative effort to assess the ice quality in relation to potential pollutant hazards to human health; with concomitant geospatial information. The geospatial distribution of ice quality and major constituents were among the measured parameters; carefully associated with further geospatial information, determined using GIS (Geographic Information Systems) and PCA (Principal Component Analysis) techniques. Interestingly, the physico-chemical analyses revealed that the ice quality was marginally adequate and the total mean metal-metalloid contents were in the sequence of Pb > Ni > Zn > Fe > Cr > As. The concentrations of these metals were above the upper allowable limits with reference to the recommended WHO guidelines. We observed that 57.1% and 35.7% ice samples had good physico-chemical properties assessed using the Ice Quality Index (IQI). Conversely, the IQI for metals showed that the ice was unsafe for human consumption. In terms of health risk assessment, the overall mean CDI (Chronic Daily Intake) and HQ (Hazard Quotient) values were in the order of Pb () > Ni (3.2) > Zn (2.3) > Fe (2.1) > Cr (1.6) > As (0.5) and Pb (7.4) > As (1.7) > Cr (0.5) > Ni (0.4 > Zn (0.008) > Fe (0.003), respectively. This study highlighted that routine monitoring of the water supplies available for making ice is required to protect public health.

## 1 Introduction

As climate change continues to threaten the globe with its disastrous consequences, Pakistan is more vulnerable among different developing countries those are suffering with yet another hike in the unprecedented temperatures [1,2]. Karachi is the Pakistan's foremost metropolis which is situated along the coastline of the Arabian Sea. Its population is over 16 million and is estimated to upsurge to 23 million within the next 15 years [3]. For the past two decades, Karachi city has experienced severe climate disasters and excessive temperature scorching >40 °C and beyond during the month of May, causing several heat waves in its wake [2]. With the long hours of electricity load-shedding and high prices making many basic necessities a thing of the past for its citizens, residents resort to different ways to find comfort in the hot weather. Unsurprisingly, with summer being the longest season, ice is considered a precious commodity that is constantly in high demand in this city. It is, thus, quite common to find makeshift ice depots doting every street in the summers, where local vendors continue to sell it at cheap rates.

In Karachi, ice is mostly consumed for edible purposes or for the preservation of edible items. One of its most frequent uses is in cold drinks, juices, milkshakes, ice-creams and Gola Ganda that are sold by the local vendors and are very popular with the masses during summers to ward off heat [4]. In addition, many also buy transparent ice due to a lack of drinking water or refrigerating appliances in their homes, which is then utilized mainly in their drinking water and other daily uses to deal with the excessive heat. Additionally, ice is frequently used to preserve the freshness of a variety of goods, particularly fish and fishery products [[5], [6], [7]] and meat products [8]. The majority of the ice used in these products is produced in local factories that use water from different sources, like tanker water, ground water and piped water supply systems, which are thought to be already polluted due to wastewater discharges from the domestic and industrial sectors. Only 1% of the industrial wastewater in Pakistan is thought to be treated before being released [9]. As a result, wastewater containing potentially harmful compounds is disposed of without considering the risks they pose to the environment. Different companies in Pakistan discharge a daily average of 40 × 10^9^ L of waste effluent into water bodies [10]. Various pollutants, like pesticides and toxic metals, have been found in drinking water sources around the country, including groundwater and surface water [11]. Heavy metal contamination is the most alarming and current trend due to extensive and varied industrial sources [12]. Entering the human body, heavy metals and metalloids have carcinogenic and teratogenic effects, offering considerable human health concerns [13]. The oral route of metals exhibited the greatest hazard index (HI) of all the exposure paths in one of the Lake Urmia assessments [14]. Therefore, the sources of water and food are the most important for the health concern of human and environment. Similarly, products made from water that will be consumed or come into direct contact with food must meet the same standards for quality and safety as drinking water [15,16]. This study, for the first time in this region, assessed the quality of the locally available ice and its potential risk for human consumption. The purpose of the study was to assess the quality of ice samples on the basis of physico-chemical parameters and metals and formulating the Ice Quality Index (ICI); and in association with the GIS spatial distribution mapping of these parameters to provide a spatial understanding of the regions with different pollution loads. The other purpose of the study was also conducting the health risk assessment of metals and metalloids in ice samples to estimate the possible hazards that would have a detrimental effects on human health. The most widely used approach—the chronic daily intake and the hazard quotient was employed for the assessment of the health risk associated with water resources. This method has been utilized in several studies conducted globally [17,18]. The physicochemical characterization and health risk assessment of metals in ice samples, a valuable commodity in Karachi, showcase the originality of this study. Transfer of toxic metals and metalloids from ice to the human body was not studied in this context prior to the implementation of this study. By using these tools, health risks related to ice samples can be found, evaluated and avoided [19]. Additionally, the most vulnerable regions in the city with high pollution levels and related health risks can be identified and producing vital information for various enforcement agencies and policy makers to improve the provision of clean ice for Karachi residents. This strategy would guide the local government to remediate the identified polluted regions, and to provide clean and affordable ice to the residents.

## 2 Material and Methods

### 2.1 Sampling

**Fig. 1:**
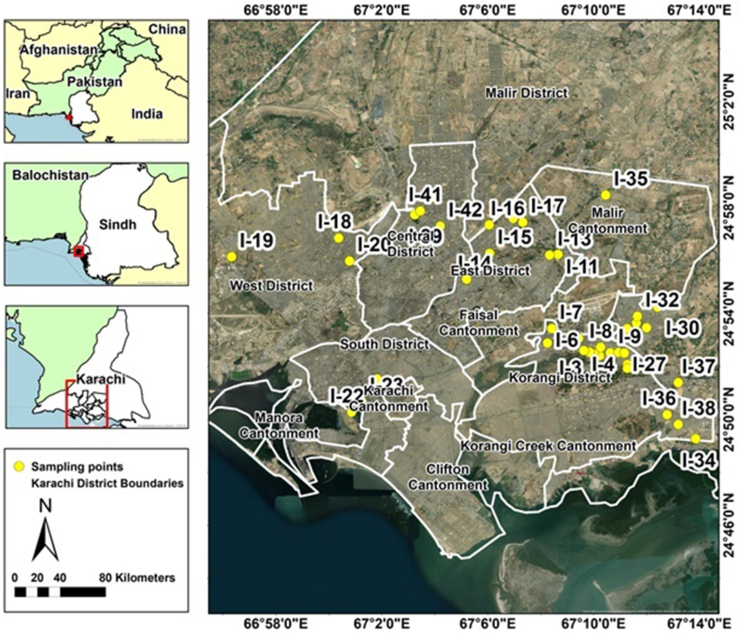
Sites for sample collection.

A total of 42 street vended ice samples were collected district wise from the Karachi city and Fig. 1 represented the distribution of sampling sites in Karachi. The samples were collected in pre-sterilized and insulated plastic containers to prevent the ice from melting and contamination. In order to transport the samples to the laboratory, they were maintained in an ice box at a low temperature. Prior to analysis, all samples were melted at room temperature and transferred into pre-sterilized glass bottles.

### 2.2 Physico-chemical analysis

The Hanna portable pH meter (HI98107) was used to record the pH of the ice samples. Turbidity of the ice samples was noted by EUTEC Turbidity meter (TN-100). TDS (Total Dissolved Solids) and sulphate of the samples were analyzed by gravimetric method. Chloride contents in the samples were measured using an argentometric method; the hardness (as CaCO_3_) in water was estimated using an EDTA titration method. Nitrate was assessed by bruccine-reagent method. The procedures outlined in APHA (2005) [20] were used to examine the aforementioned parameters.

### 2.3 Metal analysis (heavy metals and metalloids)

As, Pb, Cr, Fe, Ni, and Zn concentrations were examined in ice samples using an internationally acceptable method provided commercially through the Spectroquant Kits (Merck NOVA 60, Germany, measured optically at wavelength (nm) of 525, 525, 550, 565, 445 and 565, respectively). These test kits were pre-programmed to work with Spectroquant instruments, ensuring rapid, reproducible and precise outcomes. Furthermore, the kits were made up of validated and standard-compliant chemicals and they adhered to international standards. Spectroquant photometers worked flawlessly with all test kits. Pre-programmed calibration data were used, and an organized process with extra features that allowed us to extract the accurate results. Sample preparation and testing methods were followed according to company's protocols and manual. Further details about sample preparation and testing can be found at the website of Merck company (www.merckmillipore.com/).

### 2.4 Geostatistical and spatial distribution

The values of unknown (un-sampled) points can be determined using spatial distribution methods for interpolation based on the weighted measures and proximity-centered assumptions that closer points are more similar than the points located relatively far away. Kriging and Inverse Distance Weight (IDW) are the geo-statistical interpolation techniques that are most frequently used. Kriging is further divided into three categories: simple, ordinary, and universal. It includes adding weights to known or measured values based on how the sites where the measurements were made are oriented in space [21]. By using linear-weighted combinations, IDW, unlike Kriging, only depends on the proximity of the known (sampled) points, on the theory that closer samples points have a greater impact on the unsampled location [22,23].

Thus, using Eqs. (1), (2)), IDW is carried out for this study as follows:

(1) $$z=\frac{\sum_{i=1}^{n}x_{i}z_{i}}{\sum_{i=1}^{n}x_{i}}$$

(2) $$x_{i}=\frac{1}{d_{i}^{p}}$$

where, z is unknown value for interpolation; $z_{i}$ is *i*th data value of sampled location; n is the number of sampling points; $x_{i}$ is the weight for IDW analysis; $d_{i}$ is horizontal distance between the observed and interpolation points [24]; and p is the power of distance. ArcGIS 10.8.1 [25] IDW analysis for all parameters was carried out with the interpolation tool from Spatial Analyst ArcToolbox.

### 2.5 Statistical analysis

A Statistical software (STATISTICA, 99 Edition, Tulsa, Oklahoma) was used to examine the data collected by physico-chemical and metal analysis of ice samples in order to determine descriptive statistics for each variable. The software stated above was also used to carry out cluster analysis and principal component analysis (PCA). Cluster analysis made use of Ward's approach.

### 2.6 Ice Quality Index (IQI) Model

In this work, the ice quality metrics and their recommended WHO standards were used to compute the IQI model. The literature [[26], [27], [28], [29]] indicated that physico-chemical and metal characteristics were given a weight (w_i_) from 1 to 5 based on their significance in the evaluation of water quality for human health. As, Pb, Cr, Ni received the maximum weight of 5 in this study which is due to their greater influence on human health (Table 1).

**Table 1:** Descriptive Statistics of 42 ice samples from Karachi city.

Parameters	Districts						Overall (n = 42)	Parameters for IQI calculation		
								WHO Guideline (ppm)	Weight (wi)	Relative weight (W_*i*_)
	Korangi (n = 10)	East (n = 7)	West (n = 3)	South (n = 3)	Malir (n = 15)	Central (n = 4)				
	Mean ± SE (Min-Max) (ppm)									
pH	7.08 ± 0.17 (5.8–7.5)	6.95 ± 0.09 (6.7–7.3)	6.8 ± 0.56 (5.7–7.5)	6.9 ± 0.15 (6.7–7.2)	6.55 ± 0.21 (5.1–7.9)	6.95 ± 0.29 (6.1–7.4)	6.82 ± 0.1 (5.1–7.9)	6.5–8.5	3	0.14
Turbidity (NTU)	0.9 ± 0.56 (0.02–5.9)	0.38 ± 0.05 (0.17–0.58)	1.18 ± 0.80 (0.22–2.77)	0.42 ± 0.16 (0.16–0.72)	0.4 ± 0.073 (0.07–1.02)	1.2 ± 0.51 (0.04–2.49)	0.65 ± 0.15 (0.02–5.9)	<5	3	0.14
TDS	766.8 ± 67.7 (528–1243)	1250.71 ± 194 (762–1942)	1108.3 ± 389 (681–1886)	798.3 ± 84.07 (661–951)	880.87 ± 93.39 (512–2033)	1593.5 ± 330 (865–2451)	993.57 ± 71 (512–2451)	1000	3	0.14
Chlorides	71.17 ± 10.5 (29.99–133.9)	114.33 ± 35.25 (31.99–241.92)	94.193 ± 32.1 (40.66–151.95)	73.26 ± 6.16 (61.25–81.67)	86.21 ± 19.59 (24.5–323.89)	183.94 ± 73.7 (89.97–403.87)	96.27 ± 12 (24.5–403.87)	250	3	0.14
Hardness	217.6 ± 25.8 (116–344)	284 ± 66.11 (136–596)	389 ± 109.79 (215–592)	249.33 ± 28.8 (192–284)	286.06 ± 58.14 (124–1040)	546 ± 103.72 (284–784)	298.9 ± 29 (116–1040)	500	2	0.095
Sulphate	200.4 ± 39.8 (56–347)	208.42 ± 51.08 (56–421)	173.33 ± 82.3 (76–337)	69 ± 9.24 (53–85)	113.4 ± 22.59 (48–313)	247.5 ± 83.27 (93–432)	163.83 ± 18 (48–432)	250	3	0.14
Nitrate	3.95 ± 0.54 (1.8–6.4)	6.04 ± 1.26 (2.10–10.7)	4.36 ± 1.52 (2.6–7.4)	3.83 ± 0.26 (3.30–4.10)	4.72 ± 1.08 (1.40–14.60)	10.62 ± 3.78 (4.1–21.5)	5.23 ± 0.62 (1.4–21.5)	12	4	0.19
									∑ w_*i*_ = 21	∑ W_*i*_ = 1
As	0.113 ± 0.03 (0.02–0.33)	0.097 ± 0.019 (0.04–0.18)	0.079 ± 0.034 (0.02–0.14)	0.122 ± 0.013 (0.10–0.15)	0.12 ± 0.017 (0.03–0.26)	0.25 ± 0.032 (0.17–0.32)	0.12 ± 0.01 (0.02–0.33)	0.01	5	0.208
Pb	1.59 ± 0.285 (0.55–3.66)	1.462 ± 0.31 (0.65–2.78)	2.13 ± 0.91 (0.77–3.88)	2.65 ± 0.22 (2.32–3.09)	3.27 ± 0.44 (0.33–5.71)	3.74 ± 0.72 (1.73–5.21)	2.48 ± 0.23 (0.33–5.71)	0.01	5	0.208
Cr	0.18 ± 0.02 (0.07–0.34)	0.211 ± 0.046 (0.06–0.42)	0.21 ± 0.067 (0.10–0.33)	0.64 ± 0.07 (0.53–0.78)	0.52 ± 0.06 (0.17–0.93)	0.49 ± 0.05 (0.41–0.64)	0.37 ± 0.03 (0.06–0.93)	0.2	5	0.208
Fe	0.37 ± 0.14 (0.1–1.24)	0.461 ± 0.22 (0.08–1.62)	0.40 ± 0.22 (0.13–0.84)	0.41 ± 0.25 (0.12–0.93)	0.36 ± 0.08 (0.10–1.21)	1.56 ± 0.41 (0.39–2.19)	0.5 ± 0.08 (0.08–2.19)	0.3	2	0.083
Ni	0.62 ± 0.1 (0.21–1.21)	0.51 ± 0.11 (0.14–0.87)	0.47 ± 0.16 (0.15–0.71)	1.09 ± 0.12 (0.87–1.31)	0.93 ± 0.18 (0.11–2.55)	0.81 ± 0.23 (0.41–1.48)	0.75 ± 0.08 (0.11–2.5)	0.03	5	0.208
Zn	0.64 ± 0.11 (0.27–1.33)	0.28 ± 0.06 (0.13–0.55)	0.45 ± 0.216 (0.19–0.88)	0.33 ± 0.11 (0.12–0.45)	0.56 ± 0.06 (0.12–0.91)	0.98 ± 0.125 (0.75–1.33)	0.54 ± 0.05 (0.12–1.33)	0.5	2	0.083
									∑ w_*i*_ = 24	∑ W_*i*_ = 1

There were 3 steps in the IQI calculation. Eq. (1) was used to calculate the relative weight ($W_{i}$) in the first stage [29].

(3) $$W_{i}=\frac{w_{i}}{\sum_{i=1}^{n}w_{i}}$$

Where $W_{i}$, $w_{i}$ and *n* were the relative weight, each parameter's assigned weight, and a total number of observed parameters respectively, while the water quality rating scale ($Q_{i}$) for each of the observed water quality parameters was calculated using Eq. (4).

(4) $$Q_{i}=\frac{V_{o}}{V_{s}}X\;100$$

where $V_{o}$ and $V_{s}$ represent the observed and WHO threshold levels, respectively, for each parameter. Eq. (5) was ultimately used to determine the IQI.

(5) $$\text{IQI}=\sum_{i=1}^{n}W_{i}\;X\;Q_{i}$$

According to the WQI, water is often divided into 5 groups, as illustrated in Table 2 [[27], [28], [29]]. Unfit for drinking (WQI >300), very poor (WQI 200–300), poor (WQI 100–200), good (WQI 50–100) and Excellent (WQI 50) are the different water quality classifications. The same approach was employed for IQI classification.

**Table 2:** Ice quality index scores.

Sample code	Physico-chemical IQI	Metal IQI	Sample code	Physico-chemical IQI	Metal IQI
I-1	67.12	1547.26	I-22	40.84	4401.29
I-2	67.02	1618.35	I-23	47.32	3784.84
I-3	49.05	1666.63	I-24	39.83	2515.75
I-4	55.09	2291.89	I-25	34.46	6001.61
I-5	69.07	4286.17	I-26	42.23	5140.21
I-6	44.56	2567.64	I-27	31.32	7516
I-7	33.42	2359.8	I-28	38.65	6801.26
I-8	33.4	1636.56	I-29	31.52	7130.13
I-9	36.83	5366.59	I-30	51.94	5125.05
I-10	58.12	3025.89	I-31	51.8	4907.26
I-11	41.87	1712.17	I-32	36.34	3534.49
I-12	42.27	1487.71	I-33	43.54	2324.77
I-13	43.72	1662.03	I-34	39.65	1482.84
I-14	41.58	2263.29	I-35	119.05	840.24
I-15	91.08	1845.12	I-36	44.42	1403.22
I-16	87.42	3809.75	I-37	62.03	7575.93
I-17	100.72	2612.38	I-38	69.65	5148.70
I-18	92.08	2180.28	I-39	141.26	7442.38
I-19	45.79	1396.59	I-40	66.23	2705.89
I-20	42.91	4921.33	I-41	98.56	5525.47
I-21	41.38	3717.71	I-42	53.4	5523.86
Excellent			Physico-chemical IQI	Mean	56.39
Good				Min-Max	31.32–141.26
Poor			Metal IQI	Mean	3590.63
Unfit for use				Min-Max	840.24–7575.93

### 2.7 Human health risk assessment

#### 2.7.1 Human health risk assessment

Risk assessment is the process of estimating the possibility that an incident will occur or that exposure to environmental hazards would have a detrimental effect on the health of humans or other animals. When evaluating the health concerns associated with metals in the water, direct human ingestion is usually taken into consideration [30,31]. In this study, chronic daily Intake (CDI), reference dose (RfD) and hazard quotent (HQ) for the intake of heavy metals was calculated [32].

#### 2.7.2 Chronic daily intake (CDI)

Heavy metals and metalloids can enter the human body through a variety of pathways, such as consumption of food, skin contact, and inhalation. However, oral intake is considered to be significant compared to all other methods of ingestion [33]. The CDI (μg/(kg·day)) of heavy metal through water ingestion was calculated by Eq. (6) [32,34].

(6) CDI=CM×Lw/Wb

where, CM: metal concentration (μg/Kg) in ICE; Lw: mean rate of daily Ice intake as estimated during study (300 ml/day); Wb: body weight (72 kg) [35].

#### 2.7.3 Hazard quotient (HQ) indices

HQ evaluated the potential non-carcinogenic risks (Eq. (7); [36]. When HQ values are greater than 1, non-carcinogenic effects should be taken into account.

(7) $$\text{HQ}=\frac{CDI}{RfD}\times 0.001$$

where, CDI is the Chronic Daily Intake; RfD is the reference dose for oral toxicity. RfD values for heavy metals as described [[37], [38], [39]].

## 3 Results and discussion

Table 1 displayed the district-linkedfindings of physico-chemical and metal examinations of ice samples sold in Karachi city. According to WHO recommendations [15,16], ice that can be consumed or come into direct contact with food must meet the same standards for quality and safety as the clean water for drinking.

### 3.1 Physico-chemical characteristics

In this study, the mean pH value of 6.8 ± 0.1 was derived from all the samples. Minimum and maximum mean pH values of 6.6 ± 0.2 and 7.1 ± 0.2 were observed in District Malir and Korangi. This showed that the ice samples were neutral and towards slightly alkaline but within the WHO permissible limits.

All ice samples had turbidity that was within the range of 0.02–5.9 Nephelometric Turbidity Units (NTU), with a mean value of 0.7 ± 0.2 NTU. Both the lowest and highest value belonged to district Korangi. These measurement confirmed that the findings of the turbidity were within the acceptable range set by the WHO except one samples (Millat Town). Gross turbidity could provide an environment that is conducive for different parasites and pathogenic microorganisms to thrive [40].

TDS refers to the overall concentration of dissolved minerals in the water. WHO [16] declared that water TDS should be less than 1000 mg/L. High TDS levels in water are indicated by a change in the taste (salty). Additionally, due to an increased TDS content, severe staining or corrosion may emerge in household items and water pipelines [41]. 28.6% of the samples in this investigation had TDS levels above 1000 ppm, with a mean value of 993.6 ± 71.7 ppm. The I-39 (Shadman Town, District Central) had the highest TDS (2451 ppm), whereas Jinnah Square (Malir) (I-29) had the lowest (512 ppm) TDS contents.

Chloride in water was considered generally to be harmless for humans at low concentrations, but at concentrations greater than 250 mg/L, it would change the taste of the water. The mean chloride value in this research was 96.3 ± 12.2 mg/L. Ice samples collected from District Malir and Central have high values of chloride. Interestingly, the highest concentration of ppm was measured at the Central district.

From Table 1, 85.7 % samples were within the safe limit of hardness set by WHO. The highest hardness value of 1040 ppm was observed at I-35 (Gadap Town; District Malir). Human health would be negatively impacted by water hardness levels over 500 mg/L, which may also contribute to kidney stone production and cardiovascular disorders. High water hardness is caused due to the use of unnecessary soap and detergents.

In this study, 71.4 % samples were well within the WHO guidelines [16]. Highest level of 432 ppm was observed at District Central (Anda Mor). From Table 1, the mean district wise SO_4_ concentration were in the order of Central > East > Korangi > West > Malir > South. The overall mean concentration was 163.8 ± 18.7 ppm. Human health could be impacted by high sulphate levels (for example, by causing cancer, heart disease, and birth problems) [42].

Nitrates have significant effects in terms of human health. Nitrate levels are typically examined as part of the global monitoring of water contamination. It is possible for humans to be exposed to nitrates from a variety of environmental sources, but drinking water is thought to be the predominant source [11]. Water in Sindh and Punjab has a relatively high nitrate level [43], which is associated to agricultural runoff [44]. Additionally, several major cities in the country had reported nitrate contamination of drinking water, including Lahore [45], Islamabad and Rawalapindi [46], Quetta, and Faisalabad [47] and Kasur [48]. In this study, only two samples exceeded the safe limit [15,16]. These ice samples were collected from Gadap Town and Shadman Town, with concentrations of 21.5 ppm and 14.5 ppm, respectively.

### 3.2 Heavy Metals and Metalloids

Heavy metals and metalloids are persistent in the environment and have the capacity to bioaccumulate, which makes them an environmental threat to water bodies, soils and vegetation [[49], [50], [51], [52], [53], [54]]. The amounts of metals in ice samples have been shown in Table 1. The overall mean metal contents in all the ice samples were in the order of Pb > Ni > Zn > Fe > Cr > As. The concentrations of As, Pb, and Ni during this study exceeded the WHO recommended guidelines (Table 1). In addition, with respect to the levels of Cr, Fe, and Zn, 71.4%, 38.1%, and 54.8% of the ice samples exceeded the WHO standards, respectively (Table 1). The higher concentrations of these metals are likely to cause health problems because of the known health impacts upon consumption as a source for food, drinking, or skin contact [[52], [55]].

### 3.3 Ice Quality Index (IQI)

In this study, 42 ice samples were used to calculate IQI in 2 groups, including physico-chemical and metal parameters. Table 2 showed that, 57.1% and 35.7 % ice samples had excellent and good physico-chemical characteristics with a mean IQI value of 56.4. Conversely, all ice samples were found to be metal-contaminated with mean IQI of 3590.6 and therefore considered unfit for human consumption. The WQI of the Tanker water, sourced within Karachi city, was not satisfactory, particularly due to anthropogenic sources of pollution [56].

### 3.4 Statistical analysis

#### 3.4.1 PCA

**Fig. 2:**
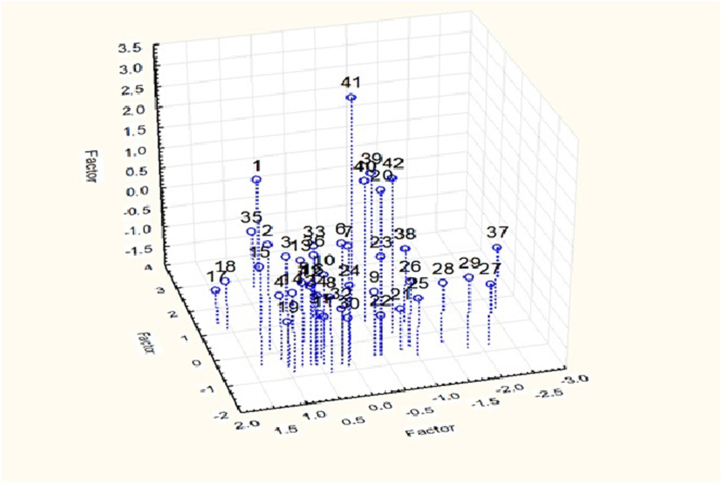
Principal Component Analysis ordination (3D) of physico-chemical and metal analysis of ice samples collected from Karachi.

The first, second and third principal components explained 30.86, 21.17 and 10.07% of the total explained variance, cumulatively 62.11% of the overall variance (Table 3). The first component is largely a function of TDS, NO_3_ and hardness. The second component is chiefly regulated by Ni, As and sulphate while the third is controlled by Fe, turbidity and Zn. The three dimensional PCA configuration is presented in Table 3 and Fig. 2. Basically, the sites with common characteristics were separated out in the 3-dimensional ordination.

**Table 3:** Results of PCA of physic-chemical and metal analysis of ice samples collected from Karachi.

Component	Eigenvalue	Percentage variance	Cumulative percentage variance	First 5 eigenvector coefficients	Associated variables
1	4.012	30.86	30.86	0.945	TDS
				0.922	Nitrate
				0.914	Hardness
				0.889	Chloride
				0.651	Sulphate
2	2.752	21.170	52.037	−0.837	Cr
				−0.792	Pb
				−0.758	Ni
				−0.651	As
				0.445	Sulphate
3	1.309	10.075	62.11	0.659	Fe
				0.657	Turbidity
				0.542	Zn
				−0.243	Chloride
				0.164	As

#### 3.4.2 Cluster analysis

**Fig. 3:**
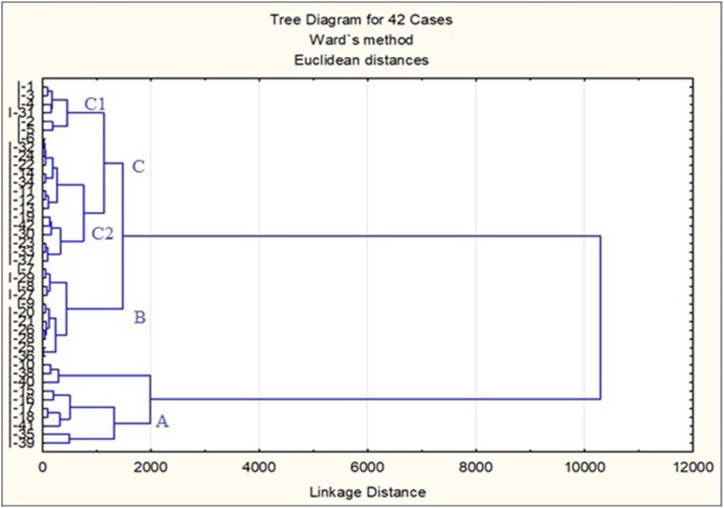
Dendrogram derived from the ward's method between 42 sites based on physico-chemical and metal analysis of ice samples collected from Karachi, Pakistan.

Fig. 3 results were obtained from Ward's clustering algorithm which is marked with three main groups. The properties of the groups were as follows;

Group A members were characterized by low to medium pH, high turbidity and TDS, medium Zn, Ni and Fe but high in Cr, Pb and As. Both SO_4_ and NO_3_ are medium. Group B sites were distributed at lower right of PCA ordination. These sites were characterized by low SO_4_ and NO_3_, low to medium TDS and high Cr and As. These sites were distributed from centre on X-axis to left side of the configuration (Fig. 3).

Group C comprised of two sub-groups C1 and C2. The group C1 appeared linearly from top left to lower space with almost neutral pH but other chemicals like Cr, PO_4_ and NO_3_ at high level although most heavy metals in low concentration (exception is Ni). Group C2 sites appeared in the lower half of PCA ordination; these were characterized by low pH, Zn and Ni and high Chloride and Cr.

### 3.5 Geospatial distribution by Inverse Distance Weight (IDW) interpolation

**Fig. 4:**
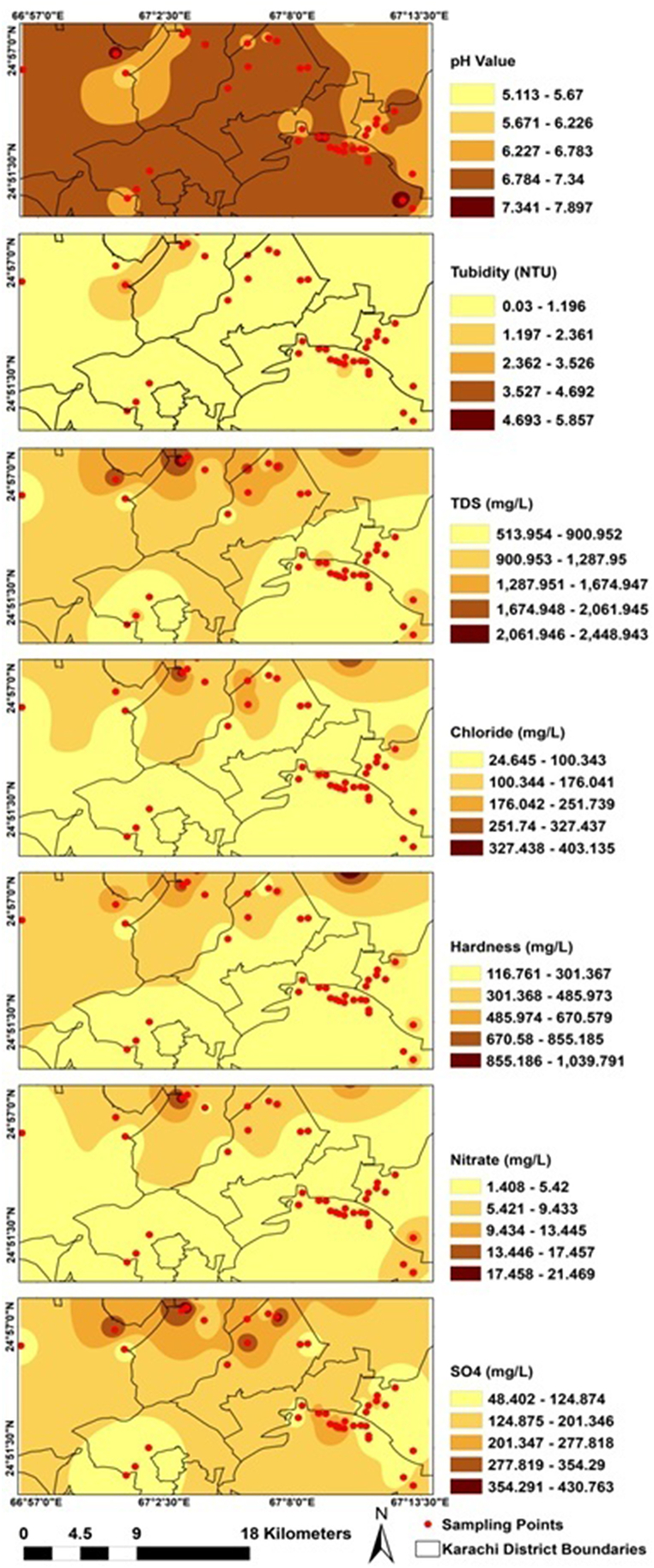
IDW based spatial distribution of physico-chemical parameters in Karachi ice samples.

**Fig. 5:**
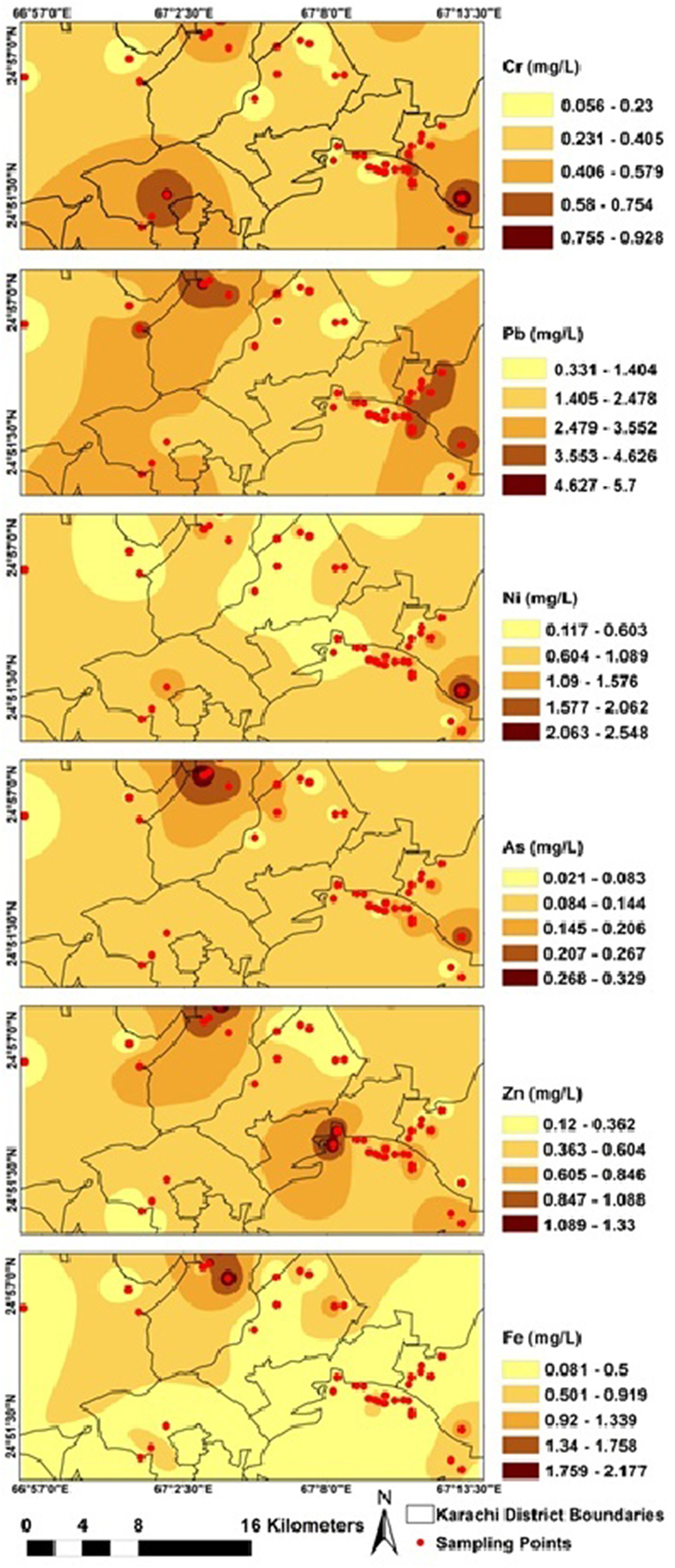
IDW-based spatial distribution of metal parameters in Karachi ice samples.

Inverse Distance Weight (IDW) was used to interpolate the geospatial distribution of physico-chemical and metals analysis as shown in Fig. 4, Fig. 5.

Many districts within Karachi had water sources that are considered as neutral to alkaline pH value except eastern part, which had partially lower pH values whereas turbidity is slightly high in the ice samples collected from northern western part. Northern western to northern eastern part showed elevated pattern of spatial distribution in terms of TDS and Hardness while northern western to north part have high chlorides and nitrate pattern. Spatial distribution patterns of ice samples in terms of sulphate were moderate values around the city. High sulphate samples were distributed from northern western part of the city (Fig. 4).

The public health situation was considered unfavourable because almost all of the sampling sites had high levels of metals (As, Pb, Ni and Cr) In the northern and eastern parts of the city, particularly high concentrations of the all metals were observed in ice samples. From Fig. 5, Elevated As and Pb concentrations were observed in the northern part and northern to eastern of the city while high Cr concentrations were observed in the southern western and southern eastern part of the city. Moreover, ice samples collected from eastern southern part of the city possessed high Ni contents. Spatial distribution patterns of ice samples with respect to higher levels of Fe and Zn were observed in the northern and northern to southern eastern part of the city (Fig. 5).

### 3.6 Health risk assessment

Table 4 showed the CDI and HQ values of ice samples collected from different districts in Karachi. In this study, overall mean CDI and HQ values were in the order of Pb(10.371) > Ni (3.156) > Zn(2.286) > Fe(2.101) > Cr(1.558) > As(0.519) and Pb(7.408) > As(1.729) > Cr(0.519) > Ni(0.405 > Zn (0.008) > Fe(0.003), respectively.

**Table 4:** CDI (μg/Kg/day) and HQ value for Metals.

District	As		Pb		Cr		Ni		Fe		Zn	
	CDI	HQ	CDI	HQ	CDI	HQ	CDI	HQ	CDI	HQ	CDI	HQ
Korangi	0.47 ± 0.35 (0.096–1.37)	1.58 ± 1.19 (0.319–4.58)	6.62 ± 3.7 (2.29–15.25)	4.73 ± 2.69 (1.63–10.89)	0.75 ± 0.38 (0.279–1.417)	0.25 ± 0.127 (0.093–0.47)	2.596 ± 1.35 (0.87–5.04)	0.13 ± 0.068 (0.044–0.25)	1.55 ± 1.9 (0.41–5.16)	0.002 ± 0.003 (0.001–0.007)	2.688 ± 1.49 (1.125–5.542)	0.009 ± 0.005 (0.004–0.018)
East	0.407 ± 0.219 (0.183–0.75)	1.35 ± 0.73 (0.61–2.5)	6.095 ± 3.4 (2.708–11.58)	4.35 ± 2.43 (1.93–8.27)	0.88 ± 0.5 (0.229–1.75)	0.29 ± 0.17 (0.076–0.583)	2.12 ± 1.20 (0.58–3.62)	0.106 ± 0.06 (0.029–0.181)	1.92 ± 2.48 (0.33–6.75)	0.003 ± 0.004 (BDL-0.010)	1.173 ± 0.724 (0.542–2.292)	0.004 ± 0.002 (0.002–0.008)
West	0.329 ± 0.248 (0.088–0.58 )	1.097 ± 0.83 (0.292–1.94 )	8.903 ± 6.62 (3.208–16.16)	6.359 ± 4.729 (2.292–11.54)	0.886 ± 0.483 (0.408–1.37)	0.295 ± 0.161 (0.136–0.458)	1.958 ± 1.2 (0.625–2.958)	0.098 ± 0.06 (0.03–0.148)	1.667 ± 1.601 (0.54–3.5)	0.002 ± 0.002 (0.001–0.005)	1.875 ± 1.563 (0.79–3.66)	0.006 ± 0.005 (0.003–0.012)
South	0.508 ± 0.098 (0.413–0.608)	1.694 ± 0.32 (1.375–2.028)	11.056 ± 1.64 (9.66–12.87)	7.897 ± 1.176 (6.905–9.19)	2.667 ± 0.53 (2.208–3.25)	0.889 ± 0.177 (0.736–1.083)	4.542 ± 0.917 (3.625–5.458)	0.227 ± 0.046 (0.181–0.273)	1.736 ± 1.860 0 (0.500–3.875)	0.002 ± 0.003 (0.001–0.006)	1.389 ± 0.771 (0.500–1.875)	0.005 ± 0.003 (0.002–0.006)
Malir	0.501 ± 0.284 (0.142–1.083)	1.669 ± 0.94 (0.472–3.611)	13.62 ± 7.23 (1.375–23.792)	9.73 ± 5.17 (0.982–16.99)	2.183 ± 1.006 (0.708–3.87)	0.728 ± 0.335 (0.236–1.292)	3.908 ± 2.958 (0.458–10.62)	0.870 ± 1.307 (0.063–5)	1.539 ± 1.4 (0.417–5.042)	0.002 ± 0.002 (0.001–0.007)	2.319 ± 1.095 (0.500–3.792)	0.008 ± 0.004 (0.002–0.013)
Central	1.042 ± 0.27 (0.708–1.33)	3.472 ± 0.9 (2.361–4.44)	15.604 ± 6.073 (7.208–21.708)	11.146 ± 4.338 (5.149–15.506)	2.063 ± 0.426 (1.708–2.667)	0.688 ± 0.142 (0.569–0.889)	3.396 ± 1.928 (1.708–6.167)	0.236 ± 0.155 (0.119–0.464)	6.500 ± 3.46 (1.625–9.125)	0.009 ± 0.005 (0.002–0.013)	4.083 ± 1.048 (3.125–5.542)	0.014 ± 0.003 (0.010–0.018)

^a^Data were presented in the form of Mean ± SD (Min-Max).

The district wise order based on mean CDI and HQ values for As, Pb, Cr, Ni, Fe and Zn were Central > South > Malir > Korangi > East > West, Central > Malir > South > West > Korangi > East, South > Malir > Central > West > East > Korangi, South > Malir > Central > Korangi > East > West, Central > East > South > West > Malir > Korangi and Central > Korangi > Malir > West > South > East respectively (Table 4).

In this study, the highest and lowest CDI values for As were observed in the districts of Korangi and West. Interestingly, district Malir had maximum and minimum CDI values for Pb and Ni, while districts East and South contributed the lowest and highest CDI values for Cr. Moreover, District Central had maximum CDI values for Fe and Zn (Table 4).

HQ > 1 in terms of As was found in 50%, 57.1%, 66.7%, and 66.7% of the ice samples that were taken from the districts of Korangi, East West and Malir, whereas HQ > 1 was present in every sample taken from the South and Central districts. In case of Pb, all the ice samples had HQ > 1 except only 1 sample (HQ < 1) collected from Gulzar-e-Hijri. Twenty percent of the ice samples in district Malir had HQ values greater than 1, while all other samples from all districts had HQ values less than 1. All ice samples collected from districts East, West, South, and Central had HQ < 1 for Ni, while 20% and 100% of the samples collected from districts Malir and Korangi had HQ > 1. Moreover, all the ice samples had HQ < 1 with respect to Fe and Zn.

These results were consistent with those of Kumar et al. (2016) [57] in India, who reported that an HQ value more than 1 indicated a sign of health danger in the human body. No possible non-carcinogenic risk for the investigated metals was found in a human health risk assessment including groundwater drinking in the Tisan River protected area [58].

Moreover, the urban drinking water samples from Qalaganj, Jiroft, and Faryab had Total Hazard Quotients (HI) of 5.06, 4.9, and 4.4, respectively, above 1. This suggested possible danger to children as well as adults [34]. Similarly, during the assessment of the topsoil surrounding Lake Urmia, the hazard index (HI) was at a safe level (HI < 1) [33]. The risks of cancer from inhaling, ingesting, and skin contact with heavy metals were shown to be negligible [33].

Furthermore, another study found that the average HQ chromium value in surface water was less than one in several Kohistan region locations [33].

### 3.7 Public health issues and contamination sources

The health effects of each parameter should be assessed in relation to the WHO recommendations when considering public health issues [15,16]. The venders usually used piped water, ground water and tanker water to prepare ice without treatment. Various investigations have been carried out in the city to assess water quality and revealed that water is grossly contaminated biologically and chemically [4,56,[59], [60], [61], [62]]. Potential sources of pollution include theft, seepage and leakage from rusty and obsolete pipelines, domestic and industrial effluents from non-point sources, inappropriate water distribution, and technical issues [4,11,56,59,60,63]. During this study, pH values of all samples were neutral and trending towards the slightly alkaline side. High amounts of bicarbonate and carbonate usually produce a pH value > 8.5. As the concentration of carbonates rises, Ca and Mg ions combine to form insoluble minerals, leaving Na ions in solution [64]. Most of the ice samples were slightly turbid which offered favorable conditions for the development of several pathogens and parasites. Some ice samples have higher levels of TDS, Chlorides and Hardness which indicates that untreated groundwater was likely used to make the ice. These kinds of ice would be harmful to public health and could cause bad taste, kidney stones and cardiovascular illnesses. Similarly, some ice samples have high SO_4_ and NO_3_ contents and they have some adverse health concerns [58]. Dehydration, gastrointestinal irritability, and catharsis are all caused by high sulphate levels. Similarly, diuresis or spleen hemorrhaging [40], Bladder and ovarian cancer as well as chromosomal damage are caused by nitrates in water [11].

Thus, the public's health may be compromised by drinking water tainted with high levels of metals, as it may have an adverse effect on their neurological, carcinogenic, and cardiovascular systems [40,58]. High metal consumption has been shown to have negative consequences on human health, and numerous studies have documented these effects. Diabetes, heart, lung and cancer illnesses can all be brought on by long-term usage of arsenic-contaminated water even at low levels [55,58]. Pb can affect multiple systems and major organs, anaemia, and deformities in humans that are irreversible due to its high toxicity, oxidative degradation, and bioaccumulative properties [[65], [66], [67]]. The prolonged exposure to lead through ice can cause headaches, sleeplessness, gastrointestinal, joint pains, fatigue, learning capacity loss, memory loss and nervous system dysfunction [40,55]. Due to its extreme toxicity, it can cause miscarriage, low birth weight and delayed development in newborns, young children and fetuses [[67], [68], [69], [70]]. Additionally, it has been observed that the bioavailable form of Cr is Cr (VI), which is highly carcinogenic when consumed. Drinking waters high in Cr can cause disorders of the liver, intestines, and stomach since Cr is cytotoxic and genotoxic to eukaryotic cells and bacteria [67,71]. Nickel has adverse effects on the respiratory and reproductive systems, as well as being neurotoxic, hepatotoxic, hematotoxic, and immunotoxic [72]. Because heavy metals and metalloids can cause cancer, Ni when mixed with Cd, Cr, and As would affect genetic materials in humans [55].

Zinc and iron are two minerals that are necessary for the human body to function properly. Fe and Zn deficiencies are common in the population of underdeveloped nations. However, an excessive use of zinc can result in both acute and long-term health problems, including anaemia, cramping, vomiting, neurological [73] and psychological [67,[73], [74], [75]].

## 4 Conclusion

This study indicated that the water supply and sewage infrastructure in Karachi are in poor conditions, inadequate, and generally obsolete in terms of functionality. The majority of the ice for consumers was prepared using untreated water. From the human health perspective, the current unsanitary conditions in the city require urgent and major infrastructural improvement. Due to engineering flaws in the sewerage system, the contaminated water was inevitably produced when household wastewater leaked into the piped water supply. This study revealed that the ice sold in the city was unfortuately polluted with heavy metals and metalloids. All ice samples were determined to be unsafe for human consumption due to the high metal contamination, with a mean IQI of 3590.6 Spatial distribution patterns of ice samples, with respect to higher concentrations of Fe and Zn, were found in the northern and northern-to-southern eastern regions of the city. The ice samples from the districts of Malir and Korangi posed severe health dangers, in to the results of the health risk assessment. Plans for water monitoring and public health education campaigns must to be employed as a futristic strategy to produce safe and clean ice for human consumption.

## Funding

No Funding Received.

## Data availability statement

The datasets analyzed during the current study are not publicly available. However, these are available from the corresponding author on reasonable request.

## Declaration

All authors have read, understood, and have complied as applicable with the statement on “Ethical responsibilities of Authors” as found in the Instructions for Authors and are aware that with minor exceptions, no changes can be made to authorship once the paper is submitted.

## CRediT authorship contribution statement

**Aamir Alamgir:** Formal analysis, Data curation, Conceptualization. **Qamar Ali:** Methodology, Investigation. **Noor Fatima:** Supervision, Conceptualization. **Moazzam Ali Khan:** Supervision, Resources. **Muhammad Farrakh Nawaz:** Writing – review & editing, Writing – original draft, Supervision. **Somia Tariq:** Methodology, Investigation. **Muhammad Rizwan:** Writing – review & editing, Supervision, Resources, Funding acquisition. **Jean Wan Hong Yong:** Writing – review & editing, Funding acquisition.

## Declaration of competing interest

The authors declare that they have no known competing financial interests or personal relationships that could have appeared to influence the work reported in this paper.
